# Development of Future Heatwaves for Different Hazard Thresholds

**DOI:** 10.1029/2019JD032070

**Published:** 2020-05-02

**Authors:** Martha M. Vogel, Jakob Zscheischler, Erich M. Fischer, S. I. Seneviratne

**Affiliations:** ^1^ Institute for Atmospheric and Climate Science ETH Zurich Zurich Switzerland; ^2^ Climate and Environmental Physics University of Bern Bern Switzerland; ^3^ Oeschger Centre for Climate Change Research University of Bern Bern Switzerland

**Keywords:** heatwave, adaptation, temperature extremes, climate projections, CMIP5

## Abstract

In 2018 and 2019, heatwaves set all‐time temperature records around the world and caused adverse effects on human health, agriculture, natural ecosystems, and infrastructure. Often, severe impacts relate to the joint spatial and temporal extent of the heatwaves, but most research so far focuses either on spatial or temporal attributes of heatwaves. Furthermore, sensitivity of heatwaves characteristics to the choice of the heatwave thresholds in a warming climate are rarely discussed. Here, we analyze the largest spatiotemporal moderate heatwaves—that is, three‐dimensional (space‐time) clusters of hot days—in simulations of global climate models. We use three different hazard thresholds to define a hot day: *fixed* thresholds (time‐invariant climatological thresholds), *seasonally moving* thresholds based on changes in the summer means, and *fully moving* thresholds (hot days defined relative to the future climatology). We find a substantial increase of spatiotemporally contiguous moderate heatwaves with global warming using *fixed* thresholds, whereas changes for the other two hazard thresholds are much less pronounced. In particular, no or very little changes in the overall magnitude, spatial extent, and duration are detected when heatwaves are defined relative to the future climatology using a temporally *fully moving* threshold. This suggests a dominant contribution of thermodynamic compared to dynamic effects in global climate model simulations. The similarity between *seasonally moving* and *fully moving* thresholds indicates that seasonal mean warming alone can explain large parts of the warming of extremes. The strong sensitivity of simulated future heatwaves to hazard thresholds should be considered in the projections of potential future heat‐related impacts.

## Key Points

- Changes in spatiotemporal moderate heatwaves strongly depend on the hazard thresholds
- We detect strong increase in projected heatwave area, duration, and magnitude if based on time‐invariant climatological thresholds
- No/minor changes are modeled in projected heatwaves when defined relative to the future climatology and changes in summer means (moving thresholds)

## 1 Introduction

Recent heatwaves such as those that occurred in the Northern Hemisphere in 2018 and 2019 have set new temperature records in multiple locations around the world (Kornhuber et al., 2019; Leach et al., 2019; NOAA, 2018; Toreti et al., 2019; Vogel et al., 2019). For instance, in France at the end of June 2019, the previous record from 2003 was broken by almost 2 °C (Mitchell et al., 2019). All‐time temperature records were also established in July in Belgium, Germany, France, Netherlands, and United Kingdom with temperatures above 40 °C in some of these regions (WMO, 2019a). Overall, July 2019 was the hottest ever observed globally (NOAA, 2019). Often, these extreme temperatures are associated with substantial impacts across diverse sectors. In summer 2018, excess mortality, crop losses, wildfires, and damage to infrastructure were reported (Vogel et al., 2019). Additionally, labor productivity is reduced during heatwaves (Dunne et al., 2013), particularly in agriculture and construction, which can lead to strong economic losses (Orlov et al., 2019). The total impacts of the 2019 heat extremes are yet to be estimated but were associated with wildfires and at least 1,500 heat deaths in France (Santé Publique France, 2019; WMO, 2019b; World Economic Forum, 2019). Thus, these recent years underline that heatwaves constitute one of the deadliest natural hazards and pose an increasing threat to human life and well‐being on all inhabited continents (Mazdiyasni et al., 2017; Singh et al., 2019). In the United States, extreme heatwaves are responsible for more deaths annually than hurricanes, lightning, tornadoes, floods, and earthquakes combined (Luber & McGeehin, 2008).

To anticipate climate change impacts associated to heatwaves, an understanding is needed of how the magnitude, spatial extent, and duration of heatwaves are projected to change. Therefore, heatwave definitions were introduced in the climate research literature, which allow studying projected changes in heatwave characteristics. Heatwaves are generally described as prolonged periods during which conditions are excessively hotter than normal, but many variations to this definition exist (Perkins & Alexander, 2013). For instance, Russo et al. (2014) proposed a magnitude index where heatwaves are defined as at least three consecutive days with maximum temperature above the daily 90th percentile temperature climatology for a reference period. Heatwave indices allow a comparison of heatwaves across regions and time but are typically limited to a grid cell level. Grid cells can be combined to consider the fraction of land covered by a heatwave but are typically not used to study the contiguous area affected by an event (Russo et al., 2014).

Considering the full space‐time dimension of heatwaves might be a more adequate way to capture their characteristics, since heatwaves develop jointly in space and time. On the one hand, the spatial component is of interest as the area of a heatwave determines the heat‐related impacts at potentially large spatial scales. The spatial extent of contiguous heatwaves impacts work capacity and reduces labor productivity, particularly for outdoor workers (Kjellstrom et al., 2009; Orlov et al., 2019). On the other hand, the duration of a heatwave can determine whether and when impacted systems can recover. For instance, longer‐lasting heatwaves can lead to stronger ecosystem impacts (von Buttlar et al., 2018). Furthermore, an increase in the duration of heatwaves can increase the mortality risk (Anderson & Bell, 2011). In combination large contiguous heatwaves in agricultural regions can influence food production, if cropping land and extensive grazing are affected. They can consequently decrease access to food and increase food price volatility (World Bank, 2012). In 2010 in Russia, an export ban led to a food price increase that affected mainly the poorest countries (Ivanic et al., 2012; Porter et al., 2014). For heatwaves that extent immensely in space and time, such impacts can even alter land use patterns (Porter et al., 2014). Power generation can be reduced during heatwaves as consequence of decreased efficiency (Añel et al., 2017). Extensive spatiotemporal heatwaves may increase demand on electricity in large regions for a long time due to higher use of air conditioning and thus could increase the risk for power outage (Perera et al., 2020).

Our current knowledge about the spatiotemporal structure of heatwaves, particularly regarding contiguous regions (e.g., neighboring countries) simultaneously experiencing heat, is very limited. A recent study investigated spatially contiguous heatwaves in the United States and shows a projected substantial increase in area, duration, and magnitude (Lyon et al., 2019). However, a global analysis of spatiotemporal heatwaves and their changes associated with different warming levels is lacking. In addition to analyzing the true three‐dimensional nature of heatwaves, the question of how we define heatwaves in a changing climate has received little attention. Generally, a fixed reference period is used when defining extremes, resulting in a strong increase in heatwaves in a warming world due to mean warming alone (Coumou & Robinson, 2013; Lorenz et al., 2010; Perkins, 2015; Zhang et al., 2011).

In our study, we investigate projected changes in moderate heatwaves at the global scale by combining a spatiotemporal heatwave characterization considering their true space‐time dimension with different hazard thresholds. We report heatwave characteristics for different levels of global warming. We then compare how moderate heatwaves evolve with climate change depending on the hazard thresholds.

## 2 Data and Methods

### 2.1 Climate Models

We use simulations from 19 different global climate models from the Coupled Model Intercomparison Project phase 5 (CMIP5) from the historical and future simulations (Taylor et al., 2012) under a high emission scenario (RCP8.5) from 1871 to 2100 (van Vuuren et al., 2011). One ensemble member is used per model. We analyze the near‐surface air temperature (*tas* in CMIP5) over land regions to compute spatiotemporal heatwaves. The complete list of models is provided in Table A1.

### 2.2 Definition of Spatiotemporal Heatwaves

**Figure 1:**
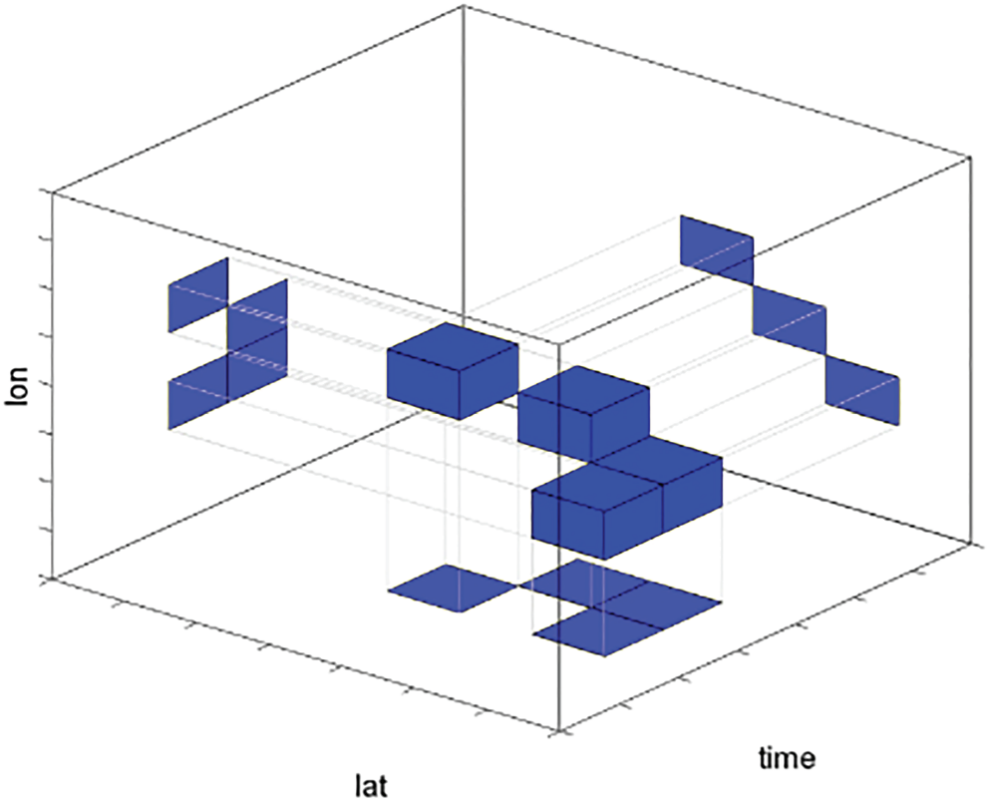
Illustration of the three‐dimensional clustering approach to define spatiotemporally contiguous heatwaves (from Zscheischler et al., 2014).

To compute spatiotemporally contiguous moderate heatwaves, we first compute the 90th percentile temperature distribution of each day based on the 31 neighboring days and 31 neighboring years. To introduce hazard thresholds, this 31‐year baseline time period is shifted. We defined three different hazard thresholds (see section 2.3) for every model simulation. Furthermore, we constrain the derived 90th percentile temperature distribution to the three consecutive warmest months in climatology (1970–2000 Zscheischler & Seneviratne, 2017). A hot day is thus defined as a day that exceeds the 90th percentile temperature distribution within these warmest months. To compute moderate spatiotemporal heatwaves, we apply a three‐dimensional clustering to the identified hot days to obtain events that are contiguous in space and time (Zscheischler et al., 2013 see illustrative Figure 1). The clusters are computed using the connected components function *connComp3D* of the *neuroim* package in R (Version 3.1.2). During the clustering, we consider all 3×3×3−1=26 surrounding cells of a given location a direct neighbor. After having clustered hot days into moderate spatiotemporal heatwaves, we only keep the 100 largest heatwaves (based on magnitude) for further analysis. We then investigate the heatwave characteristics duration, spatial extent, and magnitude.

### 2.3 Hazard Thresholds

**Figure 2:**
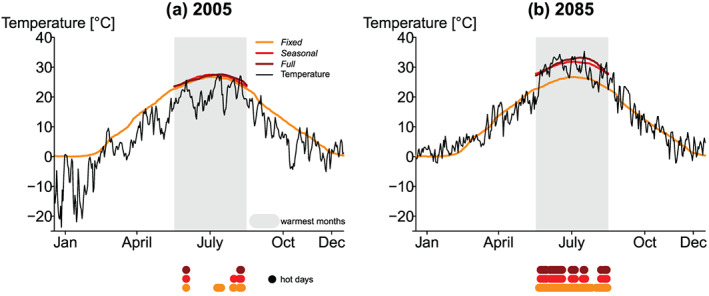
Three different hazard thresholds to compute hot days. Daily temperature (black) for (a) 2005 and (b) 2085 from CCSM4 for a grid cell close to Moscow. The *fixed* hazard threshold (orange) refers to the 90th percentile daily climatology for +0.6 °C warming. The *seasonally moving* threshold (red, only shown for the three warmest months) is based on the *fixed* thresholds plus the mean *seasonally moving* warming between 2005 and +0.6 °C (left) between 2085 and +0.6 °C, respectively (right). The *fully moving* hazard threshold (dark red) refers to 90th percentile daily climatology (based on 31 years) of 2005 (1990–2020) (left) and 2085 (2070–2100). Hot days (dots) for the *fixed*, *seasonally moving*, and *fully moving* thresholds are exceedances above the respective thresholds in the three consecutive warmest months (grey area).

We introduce three thresholds to determine hot days before we compute the spatiotemporally contiguous heatwaves: (1) *fixed*, (2) *seasonally moving*, and (3) *fully moving* (see Figure 2).

In the case of the *fixed* thresholds, hot days are computed for a fixed (time‐invariant) 31‐year climatology where warming in each model was closest +0.6 °C warming with respect to 1871–1890 (Figure 2, orange). We choose this warming level because this is the approximate warming of the period 1986–2005 with respect to 1850–1900 (IPCC, 2013).

For the *seasonally moving* thresholds, we use the *fixed* thresholds and add the seasonal warming signal. Thus, hot days are computed for a fixed baseline (such as for the *fixed* threshold) plus *seasonally moving* mean warming of the corresponding future climate based on the 31‐year moving mean of the warmest three months (Figure 2, red).

For *fully moving* thresholds, we define hot days as the exceedance of 90th percentile of the corresponding climate with a moving 31‐year temperature distribution (Figure 2, dark red). This implies that for the *fully moving* thresholds, the number of hot days stays the same over time, as the 90th percentile is computed on a moving window.

Thus, the three thresholds result in different numbers of hot days at the end of the 21st century with the highest numbers for the *fixed* thresholds (see Figure 2b). If the 90th percentile warms faster than the summer (i.e., three warmest months) mean, there will be more hot days for the *fully moving* thresholds than for the *seasonally moving* thresholds in a warmer climate.

### 2.4 Definition of Warming Levels

We compute spatiotemporal heatwaves for different warming levels. We focus on +1, +1.5, +2, and +3 °C global warming relative to preindustrial levels of 1871–1890 by applying a time‐slicing method. More precisely, for each model we select the year where the warming of a 31‐year running mean is closest to respective warming level. For each simulation we use this year as a center year for a 21‐year warming level time slice. This helps to avoid overlap between the different warming levels. Hence, we include present‐day warming, which is around +1 °C (IPCC, 2018). Global mean temperatures of +1.5 and +2 °C warming relate to the global warming targets discussed in the Paris Agreement (UNFCCC, 2015). The +3 °C warming refers to the warming that is projected to be reached at the end of the 21st century if current nationally stated mitigation ambition until 2030 will be implemented (IPCC, 2018).

Note that these warming levels are derived from transient CMIP5 model simulations and are thus relevant for climate responses within the 21st century, rather than after long‐term climate stabilization at these given levels. However, the use of transient versus long‐term equilibrated model simulations can make a notable difference (King et al., 2020).

### 2.5 Heatwave Characteristics

The spatiotemporal heatwaves are characterized by their mean area, median duration, and total magnitude. To estimate the area, we compute the mean daily land area that is affected by the heatwave over the entire duration of the event. Individual days of the heatwave can cover much larger areas than the mean area. The median duration is defined as the median duration of all cells contributing to the heatwave. The total magnitude of the spatiotemporal heatwaves is computed here as the area‐weighted sum of temperature exceedances relative to hazard threshold over all grid cells of the heatwave. To investigate changes in heatwave characteristics, we estimate empirical cumulative density distributions of the largest 100 events (based on magnitude) for the three hazard thresholds and four different warming levels. The identified spatiotemporal (three‐dimensional) events are power law distributed (Zscheischler et al., 2014), and thus, the largest 100 events from a total of tens of thousands simulated events cover on average already 45% (range across models: 36–51%) of all hot days. Hence, by characterizing the 100 largest events, we focus on the largest and presumably most impactful heatwaves.

Heatwave characteristics are presented as exceedance probabilities based on these 100 events over periods of 21 years. The exceedance probabilities thus summarize our findings over a multimodel ensemble and need to be interpreted conditioned on these 100 largest events per model. To quantify significant changes of heatwave characteristics for different warming levels, we compare the empirical cumulative density distributions of each warming level with the distributions for +1 °C with a Kolmogorov‐Smirnov test using a significance threshold of *α*=0.05.

## 3 Results

We present results on changes in heatwave characteristics for the area, duration, and magnitude. We first focus on the effect of the different warming levels for each hazard threshold and then compare the results between the hazard thresholds.

### 3.1 Heatwave Area

**Figure 3:**
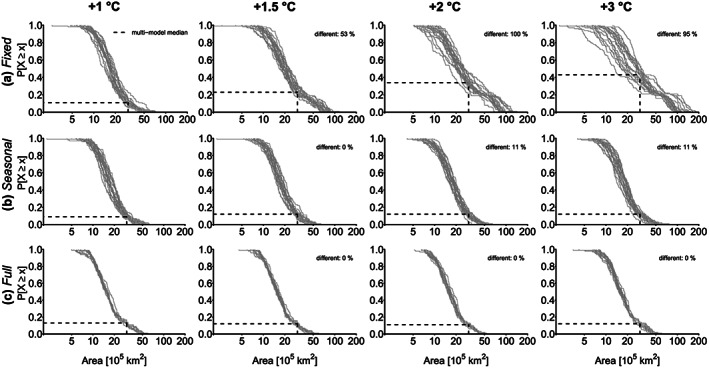
Probability of exceedance ( P[X⩾x]) for the mean heatwave area of the largest 100 heatwaves for individual model simulations (gray lines) for (a) *fixed* thresholds, (b) *seasonally moving* thresholds, and (c) *fully moving* thresholds for +1, +1.5, +2, and +3 °C global warming compared to 1871–1890. The % different indicates the relative number of models whose distributions show a significant difference (Kolmogorov‐Smirnov test, *α*=0.05) to the distributions for +1 °C warming. The dashed lines show multimodel median exceedance probabilities for an area of 3 million km^2^.

The mean heatwave area of the largest 100 events with *fixed* thresholds varies between 0.25 and 7.3 million km^2^ for +1 °C warming (Figure 3a). These areas refer to heatwaves ranging from the country size of the United Kingdom to almost the size of Australia. We find an overall strong increase in the heatwave area using *fixed* thresholds. Mean heatwave areas for +3 °C warming reach around 18.39 million km^2^ for some model simulations, which is larger than the entire land area of Russia. Given that we compute the mean area of the heatwave, on a single day, even much larger areas can be affected by extreme heat. The multimodel median exceedance probability for an event ⩾3 million km^2^ (almost size of India) increases from 11% to 43% per 100 events per model. Hence, we detect a significant difference between higher warming levels and +1 °C. For +1.5 °C more than half of the models show already significant difference to +1 °C; for +2 and +3 °C, nearly all models show significant different heatwave areas compared to +1 °C.

For the *seasonally moving* thresholds, the mean heatwave area of the largest 100 events for global warming of +1 °C can reach between 0.22 million and 6.25 million km^2^ (Figure 3b). For +3 °C warming, maximum heatwave areas can reach up to 7.69 million km^2^ (approximately size of Australia). However, the multimodel median exceedance probability (based on 100 events per 19 models) for an event ⩾3 million km^2^ only shows a small increase from 9% to 12%, and only two models (11%) show a significant difference for strong global warming of +2 and +3 °C compared to +1 °C.

With *fully moving* thresholds, the mean heatwave area of the largest 100 events ranges between 0.5 to 5.98 million km^2^ for +1 °C warming with relatively constant intermodel variability across event size (Figure 3c). This area range for single heatwaves approximately corresponds to the country size of Spain for its lowest values and exceeds the size of the whole European Union for its highest values. With *fully moving* thresholds, the heatwave area does not show any detectable change with increasing global mean temperature. The multimodel median exceedance probability for an event ⩾3 million km^2^ (approximately the land area of India) shows only a slight variation between 11% and 13%, where interestingly the highest exceedance probability is found for the lowest warming level. No significant difference between higher warming levels and +1 °C can be detected.

When we directly compare the effects of the different thresholds, we find an increasing difference in the heatwave areas for higher warming levels. For +1 °C warming, the multimodel median heatwave area is around 4% larger with *seasonally moving* and *fixed* thresholds than with the *fully moving* thresholds. For +3 °C the multimodel median heatwave area is 10% larger with *seasonally moving* and 51% larger with *fixed* thresholds compared to *fully moving* thresholds.

For the largest heatwaves, this difference is even more pronounced. For +1 °C the largest heatwave is around 4% larger with *seasonally moving* thresholds and 22% larger with *fixed* thresholds compared to the *fully moving* thresholds. For +3 °C warming, this increases to an area 27% larger with *seasonally moving* and 300% larger for the *fixed* thresholds compared to the *fully moving* thresholds. With the *fully moving* thresholds, largest heatwaves have a similar extent for all warming levels. For +3 °C warming, the largest heatwave is only 1% larger compared to +1 °C warming, 11% smaller for +2 °C compared to +1 °C warming, and equal for +1.5 °C compared to +1 °C warming.

### 3.2 Heatwave Duration

**Figure 4:**
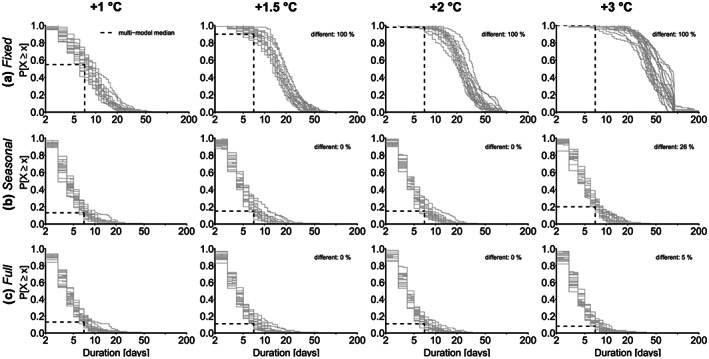
Probability of exceedance ( P[X⩾x]) for the median duration of the largest 100 heatwaves for individual model simulations (gray lines) for (a) *fixed* thresholds, (b) *seasonally moving* thresholds, and c) *fully moving* thresholds for +1, +1.5, +2, and +3 °C global warming compared to 1871–1890. The % different indicates the relative number of models whose distributions show a significant difference (Kolmogorov‐Smirnov test, *α*=0.05) to the distributions for +1 °C warming. The dashed lines show multimodel median exceedance probabilities for a median duration of 7 days.

When we use *fixed* thresholds, we find a strong increase in the heatwave duration (Figure 4a). The multimodel median duration increases from nearly 10 days for +1.0 °C warming to 47 days for +3.0 °C warming. The heatwave duration exceeds even 92 days, which corresponds to the maximum duration of the three warmest months. This can happen when heatwaves move to regions where the three warmest months are shifted to the subsequent months compared to the region where the heatwave started. As a result, heatwaves can last for several years for individual simulations at +3 °C warming. This indicates that virtually every day in the warm season is considered as a hot day. With *fixed* thresholds already for +1.5 °C, nearly every heatwave has a duration of at least 7 days. Furthermore, significant differences between +1.5 and +1.0 °C are detected for all models as well as for the higher warming levels.

With *seasonal* thresholds, heatwaves are simulated to last up to 59 days, which is slightly higher than for the *fixed* thresholds (Figure 4b). Again, differences for higher warming levels are small. The multimodel median duration increases only slightly from 4 days for +1.0, +1.5, and +2 °C warming to 5 days for +3.0 °C. The exceedance probabilities of a week‐long heatwave slightly increase from 13% for +1.0 °C, to 15% for +1.5 and +2.0 °C, to 20% for 3.0 °C global warming. However, we only detect differences between the simulated duration for +3.0 and +1.0 °C warming, where 26% of the models (five models) show a significant difference.

With *fully moving* thresholds, duration between 1 and nearly 57 days is simulated (Figure 4c). Most of the models show saturation at a maximum of around 20 days. For +1 °C the median duration can reach nearly 46 days, for +1.5 °C 57 days, for +2 °C 33 days, and for +3 °C around 37 days. The multimodel median duration is constant, with 4 days for all warming levels. Thus, we can hardly detect differences in the distributions for the increasing warming levels; only one model (5%) shows a significant difference between +3 and +1 °C warming. This is also reflected by a rather constant exceedance probability of heatwaves with a median duration of at least 7 days. For + 1°C, the multimodel median exceedance probability is 13%, +1.5 and +2 °C warming corresponds to 11% and +3 °C to 8%. Hence, already today at approximately +1 °C even with *fully moving* thresholds, every eight of the spatiotemporal heatwaves can have a duration of at least 1 week.

Again, differences for the three thresholds are most pronounced for the highest warming level.

### 3.3 Heatwave Magnitude

The simulated heatwave magnitude encompasses duration, area, and temperature anomaly and, therefore, summarizes the overall hazards of the heatwaves.

**Figure 5:**
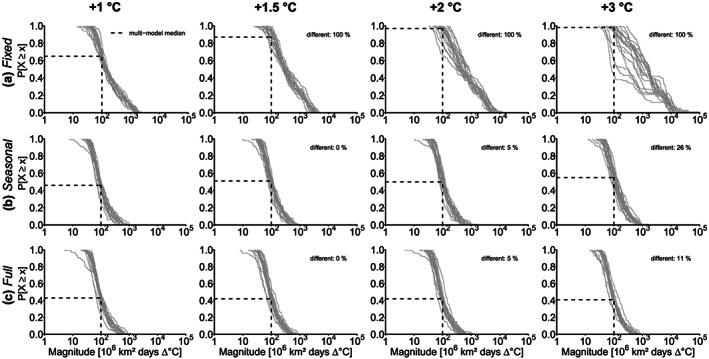
Probability of exceedance ( P[X⩾x]) for the magnitude of the largest 100 heatwaves for CMIP5 model simulations for (a) *fixed* thresholds, (b) *seasonally moving* thresholds, and (c) *fully moving* hazard thresholds for +1, +1.5, +2, and +3 °C global warming compared to 1871–1890. The % different indicates the relative number of models whose distributions show a significant difference (Kolmogorov–Smirnov test,*α*=0.05) to the distributions for +1 °C warming. The dashed lines show multimodel median exceedance probabilities for a magnitude of 100 million km^2^ days Δ °C.

With *fixed* thresholds, increasing global mean temperature is accompanied by the increasing magnitude of the heatwaves (Figure 5a). Hence, the distributions for the simulated heatwave magnitude show a significant difference for all models for +1.5, +2, and +3 °C compared to +1 °C warming. The multimodel median exceedance probability (of the 100 strongest events per 19 models) for or a heatwave of at least 100 million km^2^ days Δ °C increases from 65% for +1 °C warming to 98% for +3 °C warming.

With *seasonally moving* thresholds, the multimodel median exceedance probability for a heatwave of at least 100 million km^2^ days Δ °C increases from 46% for +1 °C to 55% for +3 °C global warming (Figure 5b). Particularly for the higher warming levels of +2 and +3 °C heatwaves exceed the magnitude of the events with the *fully moving* thresholds. For +3 °C the strongest events have a magnitude of around 1,660 million km^2^ days Δ °C. Around a quarter of the models (five models) show a significant difference in the heatwave magnitude for +3 °C compared to +1 °C warming.

With *fully moving* thresholds we again find rather constant; however, large heatwave magnitudes between 5 and 11,000 million km^2^ days Δ °C (Figure 5c). Distributions for higher warming levels do not show substantial changes. For +2 °C, only one model (5%) and for +3 °C only two models (11%) show a significant difference compared to +1 °C warming. Interestingly, the largest events for +3 °C with *fully moving* thresholds are only 55% of the magnitude of the largest event with *seasonally moving* thresholds. The multimodel median exceedance probability of a heatwave of at least 1 million km^2^ days Δ °C magnitude varies between 41% and 43% for all warming levels.

If we compare the magnitudes for the different hazard thresholds, we find that already for +1 °C warming, the likelihood of a heatwave of 100 million km^2^ daysΔ °C is much larger for the *fixed* threshold case than for the *fully moving* or *seasonally moving* threshold cases. The maximum magnitude for +3 °C reaches more than 13,000 million km^2^ days Δ °C, which is around 10 times higher than with *seasonally moving* thresholds and 20 times higher than with *fully moving* thresholds. Since the duration of the heatwaves is similar with *fully moving* and *seasonally moving* thresholds, the differences in the magnitude are mainly driven by the area and the temperature anomaly of the events.

## 4 Discussion

### 4.1 Varying Hazard Thresholds: Link to Adaptation

Adaptation to climate change is a complex process but can be an effective measure to avoid tremendous climate change impacts (IPCC, 2014). Vidal et al. (2012) introduce moving reference periods that account for different adaptation scenarios to characterize future changes in spatiotemporal droughts. These authors found that adaptation substantially reduces projected changes in droughts over France and could help understand climate variability in different possible futures (Vidal et al., 2012). We use here three hazard thresholds, which could also be interpreted as hypothetical proxies for adaptation. With time‐invariant *fixed* thresholds, we implicitly assume that adaptation may not reduce overall vulnerability to given heatwave thresholds and that heatwaves in the future will be perceived in the same way as now, independently of the experienced background mean warming. With *seasonally moving* thresholds, we implicitly account for the hypothetical adaptation to the seasonal mean warming. The *fully moving* thresholds can be seen as a proxy for full adaptation to the respective prevailing future climate. This would imply perfect adaptation with no delay to any future climate conditions.

With *fixed* thresholds, a substantial increase in area, duration, and magnitude of the moderate heatwaves is simulated, whereas no or only little changes in characteristics of heatwaves are simulated with *seasonally moving* and *fully moving* thresholds. Notably, the *fixed* threshold case with most substantial changes in heatwaves, where heatwaves can last even over the entire warm season, is at present usually assumed when changes in future temperature extremes are discussed (e.g., Coumou & Robinson, 2013; IPCC, 2012; Perkins & Alexander, 2013; Perkins‐Kirkpatrick & Gibson, 2017; Russo et al., 2016; Zhang et al., 2011). These previous studies also show an strong increase in heatwave duration and spatial extent and are thus consistent with our findings. However, such results are not surprising as in a warming world, where heatwaves are determined with exceedance above a fixed reference period, more hot days become likely. Wobus et al. (2018) additionally showed that the choice of a relative or fixed threshold largely determines future heatwave risk the in the United States. Therefore, the question arises of what should be considered as a heatwave in the future and if a fixed threshold exceedance that excludes the possibility of adaptation is always the most suitable approach.

The similarity between *seasonally moving* and *fully moving* thresholds indicates that changes in the seasonal mean warming are also driving the steady warming of extremes. This is in line with findings from other studies where the seasonal average has proven to be a useful predictor for the strong warming of extremes at higher warming levels (King et al., 2018). Gross et al. (2019) show that differences between extreme and seasonal mean warming rates are smallest for boreal summers. Consequently, if we could adapt to the warming of the warmest season, we would be able to avoid the most some of the largest heatwave impacts. Realistically, it will not be feasible to adapt to climate change instantaneously (*fully moving* thresholds) or even to the current seasonal changes (*seasonally moving* thresholds). Nonetheless, a recent study shows that the extent to which extreme weather events are perceived as remarkable by society is strongly dependent on how often these events have been experienced recently (Moore et al., 2019). These authors show that after repeated exposure to extreme events, they are not perceived remarkable anymore even though impacts can still be high. Thus, these results highlight that a moving baseline, possibly with some time delay, may describe best what societies will actually perceive as heatwaves in the future.

Our systematic assessment shows a strong dependence of the simulated changes in the characteristics of spatiotemporal heatwaves as a function of the underlying hazard thresholds. Using this analogy of hazard thresholds as proxies for hypothetical adaptation levels, this approach provides insights on how future heatwave risk might be dependent on the level of adaptation. To estimate expected changes in future heatwave impacts, changes in vulnerability and exposure and their link to adaptation levels need to be considered. Transdisciplinary efforts that include socioeconomic changes and critical thresholds of ecosystems (IPCC, 2014) would be needed to develop realistic adaptation scenarios and to eventually better estimate expected changes in future heatwave impacts.

### 4.2 The Relevance of Dynamical Changes

An increase in the duration of extreme events could exacerbate societal impacts. Therefore, it is necessary to understand if and to what extent heatwave duration could change under future warming. Changes in duration with *fully moving* thresholds would be related to physical drivers of heatwaves such as circulation changes or land‐atmosphere feedbacks. Observation‐based results suggest a trend in anticyclonic circulation patterns, a synoptic situation, which typically conducive to summer heatwaves in the midlatitudes (Horton et al., 2015). Although the presence of blocking can cause the development of persistent heatwaves (Pfahl, 2014; Röthlisberger & Martius, 2019; Zschenderlein et al., 2019), it is challenging to detect projected changes in atmospheric circulation patterns in global climate models (e.g., Shaw et al., 2016; Schaller et al., 2018; Woollings et al., 2018). There may be dynamical changes beyond the scales resolved by present‐day climate models.

However, internal variability is found to dominate the multimodel mean response of large‐scale circulations, including jet stream and stationary waves (Li et al., 2018). This is also supported by studies showing that an increase in the number of heatwave days and duration is mainly driven by the shift in the mean and not changes in variability or duration of anomalies (Ballester et al., 2010; Fischer & Schär, 2010; Lustenberger et al., 2014), for example, due to changes in synoptic conditions (Baldwin et al., 2019; King, 2019; Schaller et al., 2018).

Pfleiderer et al. (2019) investigated changes in positive temperature anomalies in atmospheric circulation models with prescribed sea surface temperatures and found a small increase in the duration of summer temperature anomalies with 2 °C global warming. The authors furthermore relate this to weakening storm track activity. We do not find evidence for a systematic increase in duration when assuming *fully moving* thresholds here. However, note that we investigate heatwaves above a 90th percentile threshold in contrast to mere positive temperature anomalies in Pfleiderer et al. (2019) and use a different set of model experiments (transient coupled rather than time‐slice atmosphere‐only model experiments). Hence, a direct comparison of the findings is not possible.

### 4.3 Future Heatwave Risk

The emerging heatwave risk can be described by changes in the (1) climatic hazard and nonclimate factors, namely, (2) exposure and (3) vulnerability (Oppenheimer et al., 2014). In this study, we investigate changes in the climatic hazards, that is, spatiotemporal heatwaves exceeding a moderate temperature threshold. Duration, area, and magnitude of such heatwaves as simulated in global climate models are large for today's warming levels for all considered hazard thresholds (e.g., a mean area larger than Mongolia, median duration more than 4 days, and magnitude more than 86 million km^2^ days Δ °C). Therefore, current heatwave risk, which would also include exposure and vulnerability, is already substantial. For future warming levels, we do not find (or only little) systematic changes in heatwave characteristics assuming *seasonally moving* and *fully moving* thresholds. Nonetheless, even if we would achieve instantaneous adaptation to climate change, the heatwave risk could still increase in the absence of an increase in the climate hazard due to increases in exposure and vulnerability (e.g., Peduzzi et al., 2012). For instance, population growth in regions prone to heatwaves can increase exposure. Russo et al. (2019) show that the projected difference in heatwave exposure will be significantly higher for +2 °C than for +1.5 °C global warming. In the United States projected exposure to extreme heat increased due to both an increase in the frequency and population growth (Dahl et al., 2019). Furthermore, changes in vulnerability can be expected. On the one hand, vulnerability to heatwaves can increase notably in developed countries due to aging populations (Anderson & Hussey, 2000). On the other hand, a decrease in human and economic vulnerability to climate‐related disasters has been observed at a global scale (Formetta & Feyen, 2019). To better estimate heatwave risk in a warmer world, it would be useful to link the identified changes in the heatwave hazard to projected exposure and vulnerability to spatiotemporal heatwaves in future studies.

## 5 Conclusions

We investigate changes in the characteristics of spatiotemporal heatwaves for three hazard thresholds under global warming. Already today, large heatwave areas, long duration, and strong magnitudes are simulated. For future heatwaves, we find a strong sensitivity in heatwave characteristics to the choice of the threshold. In particular, we show strong differences for *fixed* thresholds, that is, heatwaves that are defined relative to a historical reference period, and *fully moving* thresholds, that is, heatwaves that are expressed relative to a future climatology. What we refer to here as *fixed* thresholds is usually assumed when investigating changes in heatwaves relative to a historical baseline, and we detect a strong increase in all heatwave characteristics for this type of hazard threshold. The changes in heatwaves with *seasonally moving* thresholds and *fully moving* thresholds are relatively small, indicating that changes in the seasonal mean warming account for most of the changes in future heatwaves. For *fully moving* thresholds, no or only very few significant changes in heatwave characteristics with increasing warming levels are projected. In this context, the results suggest that thermodynamic effects alone can explain changes in heatwave duration, area, and magnitude and dynamic effects are negligible. The three introduced hazard thresholds can be seen as proxies for hypothetical adaptation levels to climate change. Given the strong sensitivity of projected heatwaves to these thresholds, this analogy underlines the active role of adaptation for projected changes in impacts from heatwaves in the future. More “realistic” adaptation scenarios that consider changes in vulnerability and exposure are needed to better estimate future heatwave risk. Also, a thorough assessment of the extent to which hazard thresholds can be affected by adaptation or not (e.g., physiological limits) is essential for such an evaluation. Such knowledge could be consequently applied for the development of effective climate risk management to abate future heatwave impacts on society, economy, and environment.

## Appendix A

### A.1

This appendix includes a supplementary table.

**Table A1:** Overview of the 19 CMIP5 Models Used in This Study

Model name	Modeling center
ACCESS1.0	Commonwealth Scientific and Industrial Research Organization (CSIRO) and
	Bureau of Meteorology (BOM), Australia
ACCESS1.3	Commonwealth Scientific and Industrial Research Organization (CSIRO) and
	Bureau of Meteorology (BOM), Australia
BCC‐CSM1.1 M	Beijing Climate Center, China Meteorological Administration
CanESM2	Canadian Centre for Climate Modelling and Analysis
CCSM4	National Center for Atmospheric Research
CESM1(BGC)	Community Earth System Model Contributors
CESM1(CAM5)	Community Earth System Model Contributors
CMCC‐CMs	Centro Euro‐Mediterraneo sui Cambiamenti Climatici
CNRM‐CM5	Centre National de Recherches Météorologiques / Centre Européen de
	Recherche et Formation Avancée en Calcul Scientifique
CSIRO‐Mk3.6.0	Commonwealth Scientific and Industrial Research Organization in collabora‐
	tion with Queensland Climate Change Centre of Excellence
EC‐EARTH	European‐Earth‐System‐Model Consortium
GFDL‐CM3	NOAA Geophysical Fluid Dynamics Laboratory
IPSL‐CM5A‐LR	Institut Pierre‐Simon Laplace
MIROC‐ESM	Japan Agency for Marine‐Earth Science and Technology, Atmosphere and
	Ocean Research Institute (The University of Tokyo), and National Institute
	for Environmental Studies
MPI‐ESM‐LR	Max‐Planck‐Institute for Meteorology
MPI‐ESM‐MR	Max‐Planck‐Institute for Meteorology
MRI‐CGCM3	Meteorological Research Institute
MRI‐ESM1	Meteorological Research Institute
NorESM1‐M	Norwegian Climate Centre

*Note*. For each model we use one ensemble member from the historical period and RCP8.5.
